# On the Feasibility of Monitoring Power Transformer’s Winding Vibration and Temperature along with Moisture in Oil Using Optical Sensors

**DOI:** 10.3390/s23042310

**Published:** 2023-02-19

**Authors:** Simplice Akre, Issouf Fofana, Zié Yéo, Stephan Brettschneider, Peter Kung, Bekibenan Sékongo

**Affiliations:** 1 Département Génie Électrique et Électronique, Institut National Polytechnique Houphouët-Boigny (INP-HB), Yamoussoukro BP 1093, Côte d’Ivoire; 2 Research Chair on the Aging of Power Network Infrastructure (ViAHT), Department of Applied Sciences, Université du Québec à Chicoutimi, 555 Boulevard de l’Université, Chicoutimi, QC G7H 2B1, Canada; 3 Modelling and Diagnostic of Electrical Power Network Equipment Laboratory (MODELE), Department of Applied Sciences, Université du Québec à Chicoutimi, Chicoutimi, QC G7H 2B1, Canada; 4 QPS Photronics Inc., 217 St. Louis, Pointe Claire, Montréal, QC H9R 5L7, Canada; 5 Département Formations Industrielles, Institut Pédagogique National de l’Enseignement Technique et Professionnel (IPNETP), Abidjan BP 2098, Côte d’Ivoire

**Keywords:** fiber Bragg grating, power transformer, winding, vibration, moisture content, temperature

## Abstract

Despite major progress in the design of power transformers, the Achilles’ heel remains the insulation system, which is affected by various parameters including moisture, heat, and vibrations. These important machines require extreme reliability to guarantee electricity distribution to end users. In this contribution, a fiber optic sensor (FOS), consisting of a Fabry–Perot cavity made up of two identical fiber Bragg gratings (FBGs), is proposed, to monitor the temperature and vibration of power transformer windings. A phase shifted gratings recoated sensor, with multilayers of polyimide films, is used to monitor the moisture content in oil. The feasibility is investigated using an experimental laboratory transformer model, especially fabricated for this application. The moisture contents are well correlated with those measured by a Karl Fisher titrator, while the values of temperature compare well with those recorded from thermocouples. It is also shown that the sensors can be used to concurrently detect vibration, as assessed by sensitivity to the loading current. The possibility of dynamically measuring humidity, vibrations, and temperatures right next to the winding, appears to be a new insight that was previously unavailable. This approach, with its triple ability, can help to reduce the required number of sensors and therefore simplify the wiring layout.

## 1. Introduction

The main function of a power transformer is to ease the versatile change in voltage and current ratios, to suitable applications. In service conditions, the windings of the power transformer are exposed to several stresses: electrical, thermal, and mechanical, even under normal conditions. Mechanical deformation to the windings can be caused by mishandling of the transformer during transportations. Another important source of deformation is related to the current in the windings. Indeed, with the increasing demand for electrical energy, the electro-dynamic forces related to overload currents or short circuit can be a serious threat to the mechanical strength and dynamic stability of the transformer winding [1].

The core and the windings mainly generate a transformer’s vibrations. Core vibration arises from magnetostriction, while winding vibration is caused by electrodynamic forces resulting from the interaction of the current in a winding with the leakage flux [2,3]. Other sources of vibration include the characteristic sound wave produced by tap-changers, and cooling system components (oil pumps and fans). One cycle of alternating current produces two peaks in the magnetic field. Suppose an AC voltage source has an amplitude that is too low (or just enough) to saturate the core, then the acceleration of the core vibrations, caused by magnetostriction of the silicon steel sheet, can be expressed by Equation (1) [2]:

(1) $$a_{c}\propto U_{0}^{2}cos^{2}4\pi ft$$

where *U*_0_ represents the amplitude of the applied voltage, and *f* the frequency.

The vibration forces, *F_w_*, are proportional to the square of the load current *I*, and are given by Equation (2) [2]:

(2) $$F_{w}\propto I^{2}$$

Since the electromagnetic forces are proportional to the acceleration, *a_w_*, of the windings, it yields [2]:

(3) $$a_{w}\propto I^{2}$$

The core vibration frequency of the windings is twice that of the alternating current. The difference between the two types of vibration is that the core vibration amplitude is based on the voltage applied to the primary windings and is not affected by the load current, while the winding vibration amplitude is proportional to the square of the load current. However, the vibrations of the core can be neglected in comparison with the vibrations of the windings in the case of short-circuit tests. In addition to the voltage and current that cause or induce vibration in the transformer, temperature, power factor, and internal faults can also influence vibrations [2,4,5].

According to some studies, 12% to 21% of transformer failures are caused by winding deformations [3,6,7]. The vibrations generated by the core and the windings propagate through the insulating oil to the transformer tank [2,4,8,9].

Investigations on electromagnetic vibrations in transformers began in the 1920s, with limited resources [10]. According to Kaixing Hong et al. [9], the diagnostic techniques of a vibration signal can be divided into two groups: signal based and model based. In recent years, some models were developed to monitor the mechanical integrity of a power transformer’s core and winding vibrations. Belén García et al. [3] developed a model for a transformer monitoring system to estimate the tank’s vibration. Hong Zhou et al. [11] developed a winding vibration model coupled with an electromagnetic force analysis to obtain the steady state vibrations in the axial direction. Since electrical substations in which power transformers are installed are more and more commonly in urban areas, the noise generated by power transformers may be masked, and hence difficult to characterize. Bo Zhang et al. [12] proposed a novel approach to investigate the core vibration in power transformers. According to these authors, the iron core is the main source of noise among the noise sources in a power transformer.

To avoid potential catastrophic consequences, different methods allowing the monitoring of the physical integrity of the transformer such as: the frequency response method (FRA) [13] sweep frequency response analysis (SFRA) [14], leakage reactance method [15], and winding deformation analysis, in power transformers using the Finite Element Method [10] were proposed. Although these methods are effective in studying the physical integrity of the transformer, they do not provide real time monitoring of the power transformer windings’ integrity, to reflect dynamically their condition [16].

Two other threatening parameters inside transformers are moisture and temperature. Moisture in a transformer may arise from various sources [17]:

- Insufficient drying at the manufacturing site;
- Exposure to humid air during site installation/commissioning or maintenance;
- Aging of the cellulose produces water;
- Leaking gaskets;
- Malfunction of the dehydrating breather;
- etc.

Traditional measurement techniques of moisture in oil are reported in the literature [18]. Among others, the Karl Fisher titration remains the most widely accepted laboratory technique. In recent decades, capacitive probes, consisting of two electrodes with a dielectric made by a hydroscopic polyimide, have emerged for online moisture assessment [19]. Polyimides have weak conductive and capacitive dielectric properties. Hence, moisture absorbed by polyimides immersed in the oil, is reflected in the capacity, owing to the high relative permittivity of water at room temperature (80), compared to that of transformer oil (2.2) [20]. This is because the influence of ageing byproducts—having a low permittivity compared to water—is negligible.

Yao, and other researchers, also developed temperature and humidity based polyimide-coated FBG sensors [21]. Lin et al. [22] reported that the Bragg wavelength of the polyimide-coated FBG is sensitive to different humidity conditions. This is because the polyimide coating expands with humidity absorption.

Moisture particularly affects the dielectric characteristics (conductivity, breakdown strength, dissipation factor, etc.), along with the resistance to ageing, of the oil-paper insulation [23,24,25]. The insulation ageing is catalyzed by heat, through pyrolysis, ageing by-products, dirt, vibrations/overload, and electrical stress/voltage waves, etc.

With the growing interest in smart grids, online monitoring systems are seen as a panacea. The aim of this paper is to propose a fiber optic sensor (FOS) technique with the triple ability to monitor the temperature and vibration of a laboratory transformer winding, in addition to the moisture content in the oil. Unlike purely electrical transducers, FOS are immune to electromagnetic fields, are relatively smaller in size, corrosion-resistant, easy to be miniaturized and multiplexed, and benefit from a long experience in the telecommunications industry [26]. This sensor consists of phase shifted gratings recoated with multilayers of polyimide, which forms the sensor, fitted inside a spacer between two neighboring windings. A temperature probe is inserted into the transformer tank near the FOS to provide a baseline for comparison. The moisture content, measured with the optical sensor, is correlated to that obtained with the Karl Fisher titrator from oil taken from the laboratory transformer. The vibration signal of the FOS is compared with the signal collected from accelerometers. 

## 2. Materials and Methods

The methodology for these investigations, is divided into two parts: first, the measurements are performed on a laboratory winding model; secondly, the results are analyzed and discussed.

### 2.1. Fiber Bragg Grating (FBG) Vibration Model

Optical fibers are, by nature, insensitive to electromagnetic fields, but transmit external environment parameter variations. Therefore, when a beam of light passes through an optical fiber, external vibrations can be sensed because of the photoelectric effect [9]. This is because the amplitude, phase, wavelength and polarization of the optical-electric field, are concomitantly affected while the sensed signal propagates in the fiber. A signal reversing method can then be applied to monitor vibrations [20]. 

With FBG interference technology, optical fibers make it possible to design sensors capable of operating inside power transformers. The sensor has been fabricated using material that can support extended working temperatures, up to 150 degrees Celcius. According to the basis of microdisturbance-scale light coupling mode theory, the effective refractive index of the lattice region, and the center wavelength, of the FBG can be obtained by solving Equation (4) [27]:

(4) $$\delta n_{eff}=\bar{\delta n_{eff}}[1+cos\left(\frac{2\pi }{\Lambda }z\right)]$$

where $n_{eff}$ represents the effective refractive index, Λ the grating period, $\bar{\delta n_{eff}}$ the variation of the average refractive index, and z the axial direction.

The Bragg (central) wavelength, $\lambda_{B}$, that interacts with the undulation of the optical fiber, is represented by:

(5) $$\lambda_{B}=2n_{eff}\Lambda$$

Since the refractive index of the fiber varies with strain along the axial direction (Equation (4)), and assuming that the grating is only affected by this later, the central wavelength shifting value of the FBG can be obtained as follows:

(6) $$\frac{\Delta \lambda_{B}}{\lambda_{B}}=\frac{\Delta \Lambda }{\Lambda }+\frac{\Delta n_{eff}}{n_{eff}}$$

where Δ*λ_B_*, ΔΛ, and Δ*n_eff_* represent the variation of the FBG’s central wavelength, the grating period, and the refractive index, respectively. The shift in the Bragg wavelength can be expressed as a function of the relative humidity and temperature [22]:

(7) $$\frac{\Delta \lambda_{B}}{\lambda_{B}}=K_{T}\Delta T-K_{H}\Delta H$$

where *K_T_* and *K_H_* are the temperature and moisture sensitivities, respectively, of the material used to coat the FBG. For the FBG coated with material not sensitive to moisture, Equation (7) simplifies to:

(8) $$\frac{\Delta \lambda_{B}}{\lambda_{B}}=K_{T}\Delta T$$

### 2.2. Fiber Bragg Grating (FBG) Temperature Model

Any change in the temperature is reflected in the central wavelength, which is shifted based on Equation (5). With the assumption that the fiber grating is only affected by the temperature, the central wavelength shifting value of the FBG can be obtained [25]:

(9) $$\frac{∆\lambda_{B}}{\lambda_{B}}=\left(\alpha_{s}+\varsigma_{s}\right)\Delta T$$

where the grating pitch variation with temperature is described by αs=1ΛΔΛΔΤ, which depicts for the fiber’s thermal expansion coefficient.

The FBG’s thermo-optic coefficient, given by Equation (9), describes the refractive index changes with temperature:

(10) ζs=1neffΔneffΔT

The variation of the ambient temperature is reflected in the central wavelength, Δλ*_B_*, according to Equation (7).

**Figure 1:**
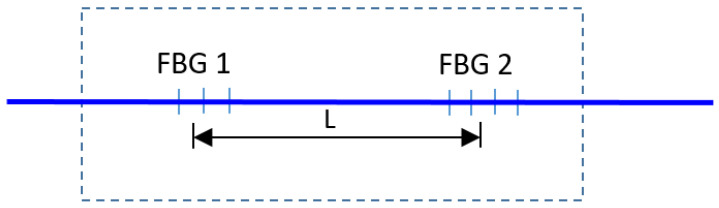
Schematic of the fiber optic sensor.

A deep ultraviolet laser and a phase mask technology (one of the most effective FBG methods), were used to transfer a periodic pattern into the core of a single mode photosensitive optical fiber [28]. The fiber recoater S541A, by Furukawa Electric corp., was used for both acrylate FBGs. The vibration and temperature sensor are obtained by printing two different grating FBG structures, placed at a small distance from each other, on the same fiber, thus forming a cavity (Figure 1).

This sensor measures wideband vibration signals spanning 2 Hz to 2 kHz. The signal is then transmitted to the interrogation unit, for further analyses. When a corresponding central wavelength laser beam is sent into the cavity, it is reflected to a π phase, inducing two interferential beams and a dense fringe. The stimulating source of the incident light consists of an incident optical source. 

A longer cavity length induces a dense fringe pack with a steeper slope, affecting the detection sensitivity. The vibration function is achieved by programming an operating point in the middle of the upward slope of a selected strip [29].

**Figure 2:**
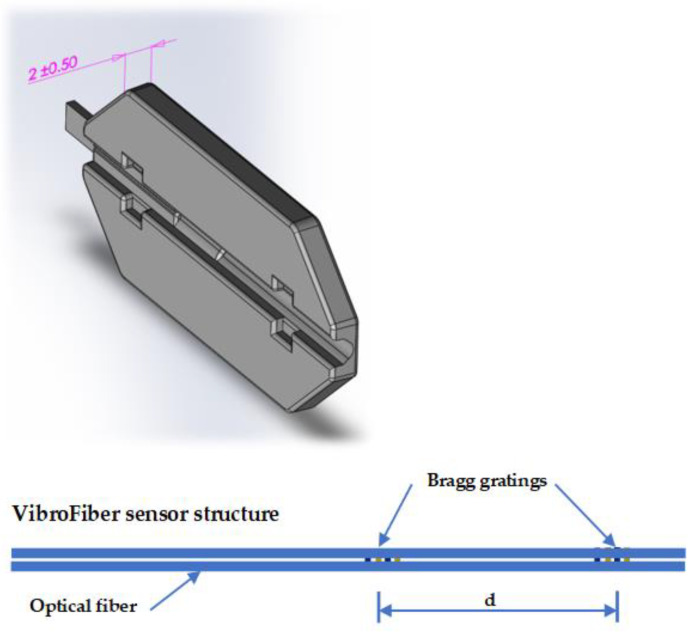
The designed sensor (30 mm × 14 mm × 2 mm).

When a vibration occurs, the fringe pattern moves right and left, forcing the operating point to oscillate the slope, which is translated into linear intensity changes. The change directly reflects the actual vibration in the winding. The architecture of the sensor is housed in a 2 mm thin package (Figure 2).

**Figure 3:**
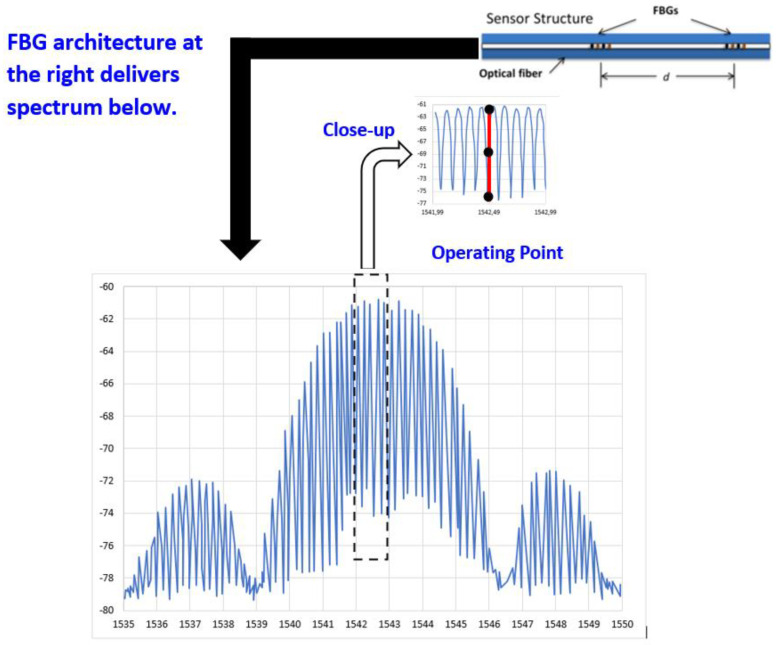
Reflection Spectrum for a fiber optic vibration sensor.

The FBG’s cavity reflection spectrum, showing all fringes, is given in Figure 3.

This reflection spectrum consists of interference fringes within an envelope. It corresponds to the reflection spectrum of each individual FBG.

By programming an operating point at the midpoint of the rising slope of a selected fringe, the vibration function is realized. Under vibrations, the fringe pattern moves right and left, forcing the operating point to move up and down the slope. This is translated into linear intensity changes that accurately reflect the actual vibration. To discriminate the influence of temperature, a self-calibration algorithm was introduced to re-establish the operating point, and such compensation delivers an indirect method to monitor temperature [25].

Since transformers usually emit vibration signatures ranging from DC to 1 kHz, a shaker (YE5503), producing vibration stimuli varying from 20 Hz to 1 kHz, was used to calibrate the sensor.

### 2.3. Fiber Bragg Grating (FBG) Moisture Model

To provide an additional possibility for moisture assessment, the vibration sensor structure was modified. Coating with moisture sensitive polymers was required. Polyimide was selected because of its sensitivity to humidity due to the unique hygroscopic properties, good electromagnetic, thermal and mechanical stability, and wide application in capacitive probes [30].

From Equation (7), it can be seen that the Bragg wavelength of the polyimide-coated FBG changes with humidity variations, due to the volume expansion of the coating. According to Lin et al. [22], the moisture sensitivity of the polyimide-coated FBG is mainly influenced by the thickness of the coating and the radius of the fiber. Hence, variations of the vapor pressure of water in oil affect the pressure on the polyimide films. Buck proposed an empirical equation to model the vapor pressure of pure water in oil [31]:

(11) $$p_{o}=6.1121e^{\left(\frac{17.502\;\times \;T}{240.97\;+\;T}\right)}$$

This indirect pressure from the moisture absorption can be monitored by the resonance wavelength shifts. With moisture increase, the material shrinks and compresses the fiber, hence causing a reduction in its center wavelength for an accurate ppm measurement.

**Figure 4:**
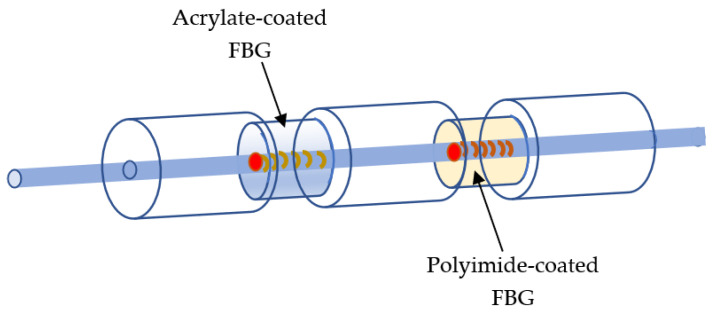
Schematic of the designed humidity sensor.

**Figure 5:**
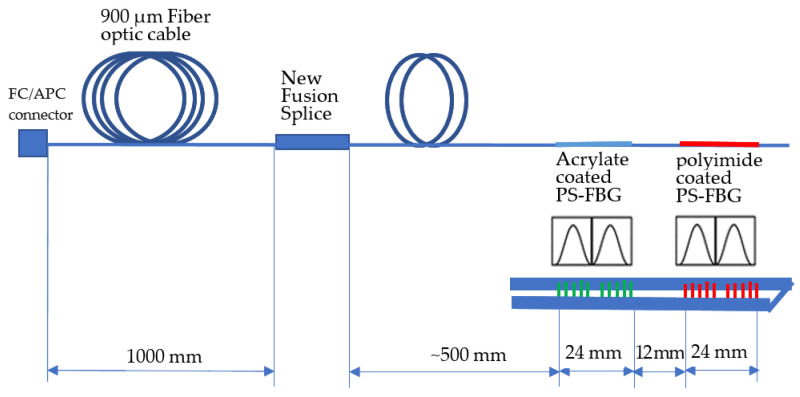
The ppm moisture sensor architecture and its connection.

**Figure 6:**
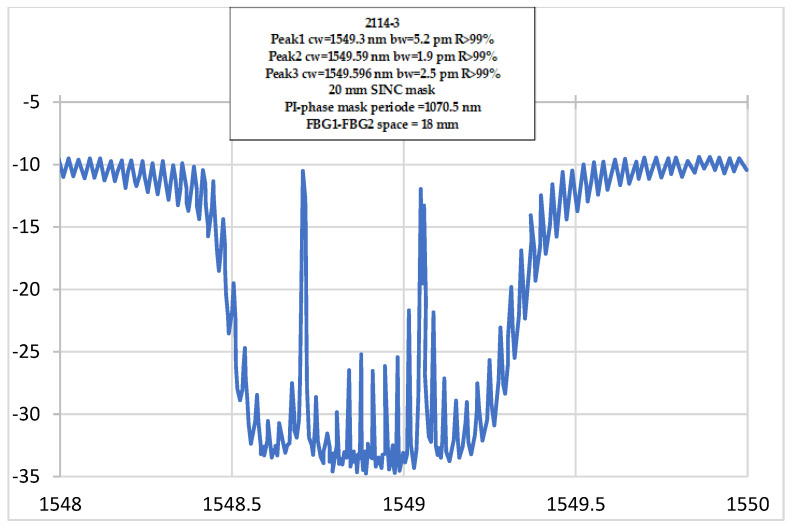
The smaller peak measures the ppm moisture, the larger peak measures temperature only.

Two different FBGs were jointly placed (Figure 4 and Figure 5). While phase shift gratings with conventional acrylate coatings depend only on the temperature, vibrations, and moisture, polyimide films, which depend on temperature and moisture, were used to coat the phase shift gratings during curing at 300 °C. The automated polyimide fiber recoater PRL201, by Vytran, was used. Because both FBGs are very close, and experience the same vibrations and temperature (the same external condition), the difference between peaks allows an assessment of the moisture. The distance between the two peaks (Figure 6) is directly calibrated to ppm of moisture, measured by Karl Fisher titration [29], taking into account the moisture solubility in oil, which is temperature dependent.

The separation between the narrow peaks in the composite plot is largest when there is no moisture. It relates inversely to ppm value.

## 3. Laboratory Setup

**Figure 7:**
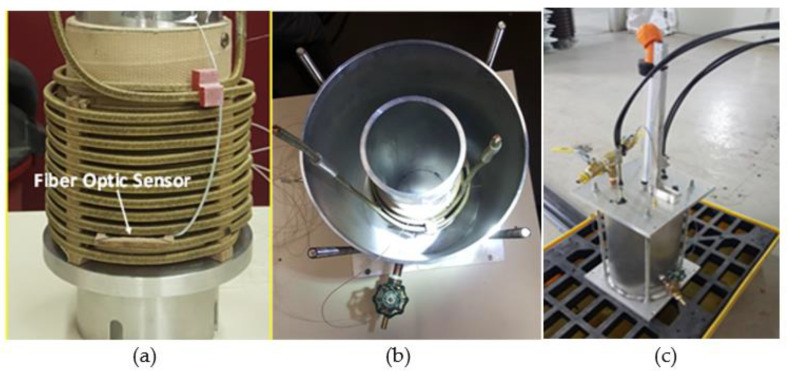
Laboratory transformer: (**a**) overview of the core, pressed paper, and winding assembly; (**b**) overview of the tank containing the core winding assembly; (**c**) overview of the laboratory transformer with external connections.

To test the FBG fiber optic sensor, and verify its ability to operate in a real-life power transformer (TP), a laboratory oil-filled transformer was designed and fabricated (Figure 7).

**Figure 8:**
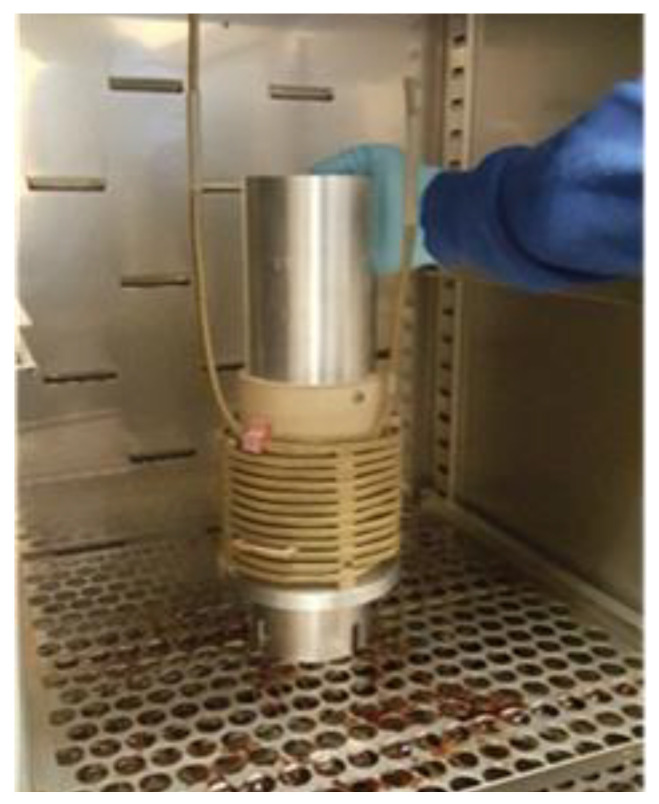
Core and winding assembly, in the vacuum drying oven.

The laboratory transformer, filled with insulating oil, consisted of a cylindrical core (Figure 7a), equipped with a pressboard, on which a copper wire winding was made. The tank was hermetically sealed by a cover on which various hardware was mounted (Figure 7c). The laboratory transformer was oven-dried at 105 °C under vacuum, for 48 h, to remove moisture. It was then filled with Nynas brand new mineral oil (vacuum degassed for 48 h). Figure 8 shows the photograph of the core and winding assembly, in the vacuum drying oven. The temperature given by the FOS is compared with that given by a high temperature thermocouple (TJ 36-CASS-116G-12-CC) installed near the fiber optic sensor, close to the winding, inside the laboratory transformer.

**Figure 9:**
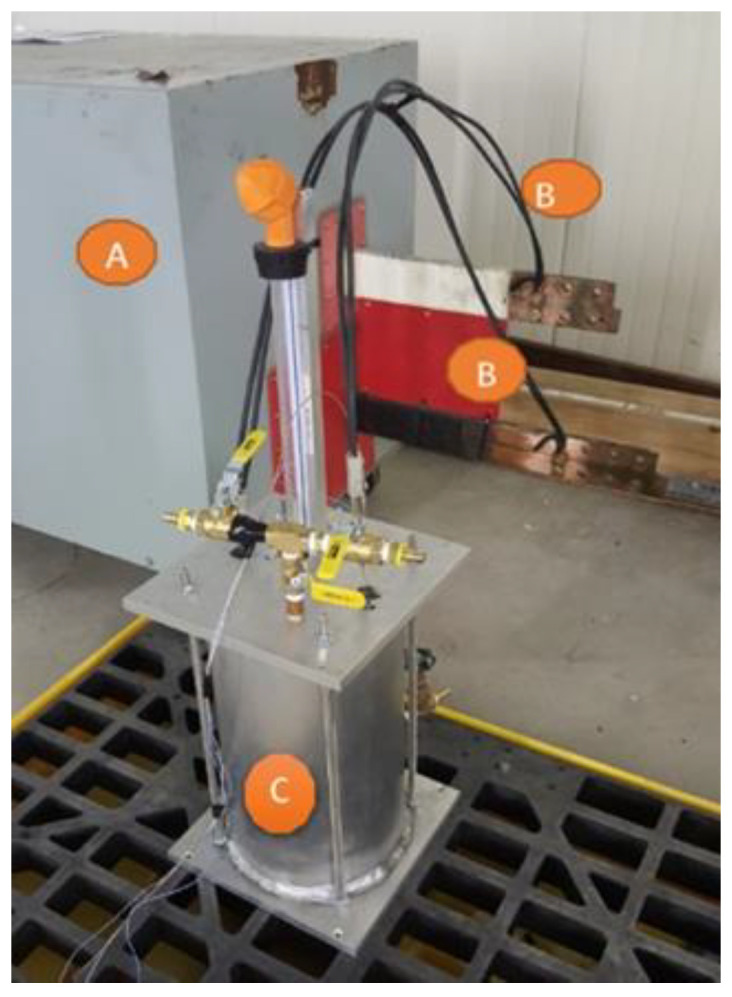
Experimental transformer connection to the high current source. (A) High current source CIGELE-S5000A, (B) Connection cables, (C) Laboratory transformer.

**Figure 10:**
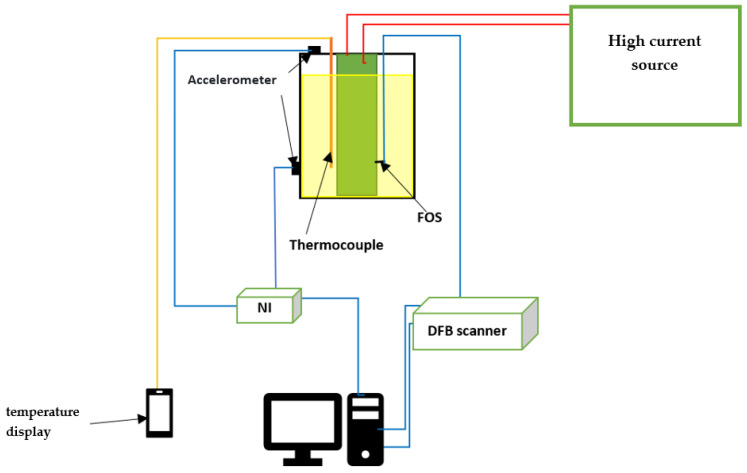
Schematic of the experimental setup (FOS: Fiber optic Sensor).

**Figure 11:**
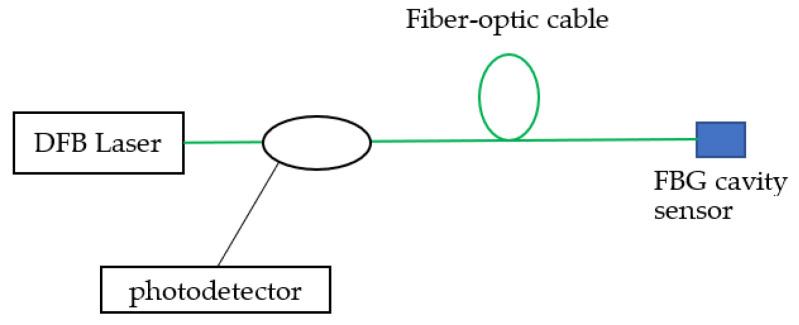
The distributed vibration sensor method.

Figure 9 shows the laboratory winding model and its connection schematics. A low-output voltage transformer, allowing the delivery of a high current, up to 3500 A, was used. Two accelerometers were used to monitor the vibrations. One accelerometer was placed on the tank and the second one on the cover (Figure 10). A national instrument, NI DAQ-9174, was used to collect and store the data from the accelerometers. A DFB scanner was used to record the data from the FBG, and all data were saved onto a computer. A photoelectric converter allowed the conversion of the output optical signal into an electrical one. This was later transmitted to the computer, and processed through a specifically written program (with graphical user interface), to determine the temperature and vibration (Figure 11).

## 4. Results and Discussion

### 4.1. Load-Dependent Vibrations

**Figure 12:**
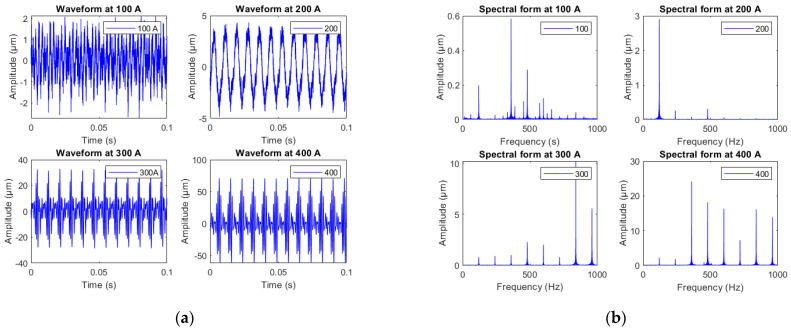
Influence of the loading current on the winding vibrations. (**a**) temporal waveforms of the vibration, (**b**) spectral forms of the vibrations.

To simulate different operating conditions, the winding current was varied from 100 to 400 A. Figure 12 shows some tests performed to assess the effectiveness of the FBG in effectively monitoring the vibrations in the windings. As expected, when the load current increases, the amplitude of the winding vibrations increases too. Indeed, vibrations in the windings are generated by the Lorentz force, caused by the interaction between dispersed magnetic flux and electric current [8]. This result means that the amplitude of the vibrations is proportional to the applied current. The Lorentz force leads to an oscillation with doubled electrical frequency. In addition, the magnetic leakage flux increases, causing magnetostriction in the leakage flux traps, which induce additional vibrations. The waveform’s change from 100 A to 400 A is therefore affected by superimposed frequencies originating from the mechanics of the transformer model. Indeed, at a given load condition, the effects of the current superimpose with magnetostriction [32].

The results are also in agreement with those reported in the literature, indicating that vibrations with frequencies greater than 100 Hz are related to the core [9].

### 4.2. Temperature Monitoring with the Sensor

**Figure 13:**
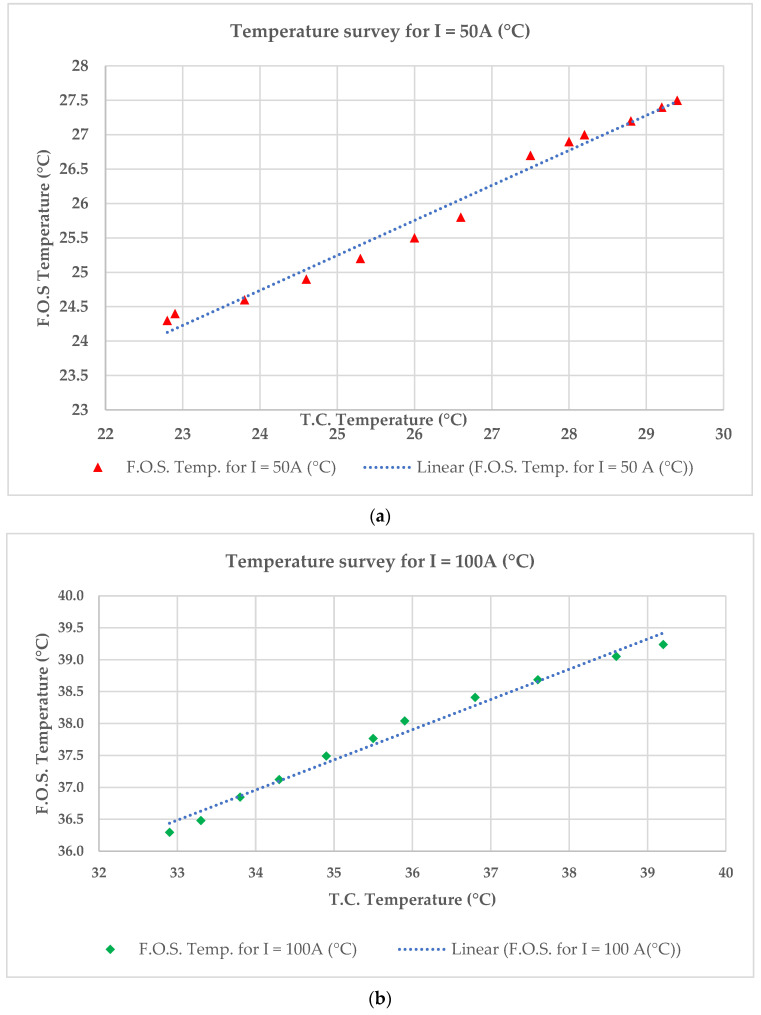

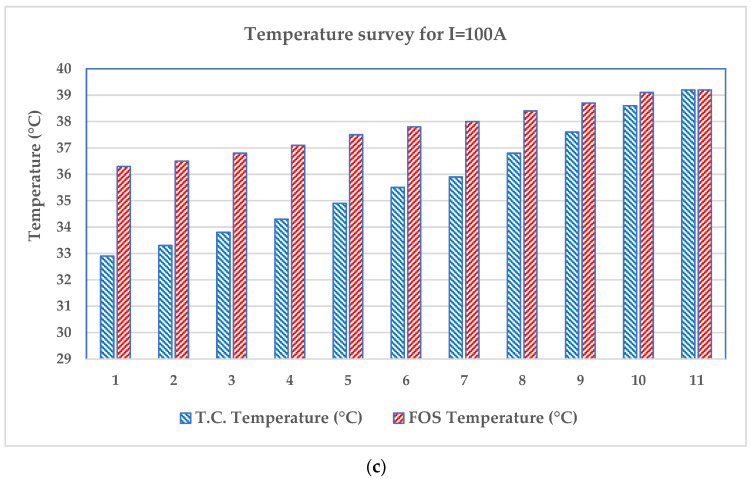
The temperature measured by the thermocouple and the FOS for two different values of intensity, (**a**) I = 50 A, (**b**) I =100 A and (**c**) comparative values of temperatures provided by the FOS and thermocouple for I = 100 A.

The temperature measured by the thermocouple (T.C.) and the FOS for two different values of intensity, i.e., 50 and 100 A, are presented in Figure 13a,b, and are compared for I = 100 A in Figure 13c.

The difference between the temperature given by the thermocouple and the FOS varies between 0.1 °C and 1.9 °C. So, the relative error between the thermocouple and the FOS varies from 0.4% to 6.91% for I = 50 A. For I = 100 A, the difference between the measured temperatures is around 3.4 °C and, therefore, the maximum relative error is around 9.36%. It should be mentioned that the relative error between the temperature provided by the thermocouple and the FOS is mainly because both probes are not placed at the same position. Generally, the FOS is often closer to the heat source (the windings) than the thermocouple, and therefore the temperature values provided by the FOS are slightly higher than those of the thermocouple. 

### 4.3. Moisture in Oil Assessment

**Figure 14:**
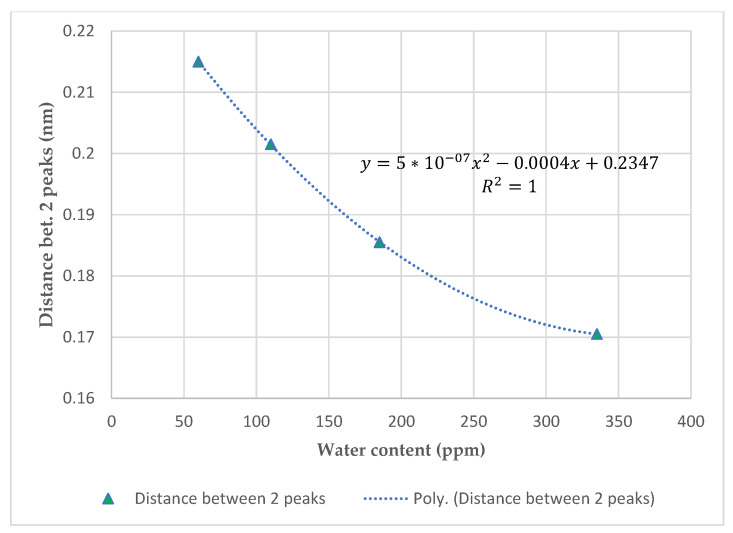
Wavelength domain feedback curves for different exposure times to Midel 7131 water content after insertion of FOS.

**Figure 15:**
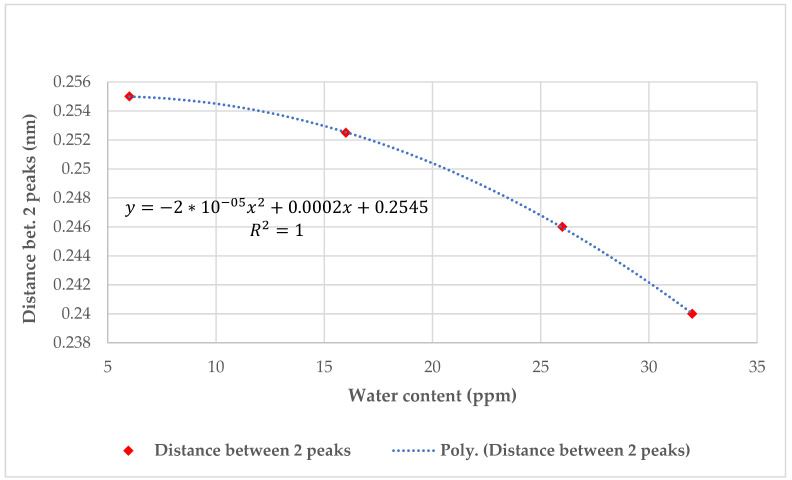
Wavelength domain feedback curves for different exposure times to MO water content after insertion of FOS.

To assess the effectiveness of the moisture assessment, regardless of the type of the fluids, a mineral oil and a synthetic ester were considered. The samples were conditioned at different moisture contents based on Karl Fisher titration (KFT). In order to vary the moisture level, the insulating fluids were exposed to saturated humid air under constant stirring [33]. The exposure was stopped after different durations, to obtain four different moistened fluid samples. A careful anal ysis of the signals recorded from the sensor allowed the correlation of the absolute moisture in ppm to the distance between both peaks. Figure 14 and Figure 15 show the moisture content assessed by KFT and the sensor output (distance between two pics), respectively, for synthetic ester oil 7131 and the mineral oil. The perfect correlation factors indicate the feasibility of the approach. However, both fluids depict different behavior, indicating the influence of the chemical compositions. The authors will engage in a wide range of investigations, including the influence of aging, byproducts, and temperature to guarantee real life applications.

Figure 15 presents the moisture content in mineral oil (MO) after insertion of the FOS.

## 5. Conclusions

It is a well-known fact that vibration, temperature, and moisture are serious problems for large power transformers. Monitoring these parameters is helpful to ensure the health of these important machines, considered as the heart of any power grid. In this contribution, an optical sensor is proposed to measure these three parameters at the same time. 

The vibration and temperature sensors are obtained by printing two identical conventional acrylate coated FBG interference networks at close distance from each other, on the same fiber, thus forming a cavity. For moisture assessment, polyimide films were used to coat one of the FBGs of a companion sensor. An experimental one-winding transformer, specially designed for this purpose, was used as the testing equipment. The temperatures monitored by the sensor, right next to the winding, compare well with those collected from thermocouples. The sensor also shows capability in monitoring vibration, as assessed by sensitivity to the loading current. The correlation of the distance between the two peaks of the moisture sensor indicates a very good agreement with absolute moisture in the liquid.

The authors are still engaged in further experiments to assess the influence of the type and condition of the fluid in the moisture, along with the sensitivity in detecting defective windings. Above all, this work can be seen as a benchmark for monitoring, at the same time, humidity in the oil, vibrations, and temperature, right next to the winding.

## Data Availability

Not applicable.
